# Multi-Scale Performance Assessment of Asphalt Mixtures Modified with Waste PVC Foils of Varying Geometries

**DOI:** 10.3390/polym18080993

**Published:** 2026-04-19

**Authors:** Ufuk Kırbaş, Erol İskender, Tuba Kütük-Sert, Faridullah Hayat, Sezai Kütük

**Affiliations:** 1 Department of Civil Engineering, Faculty of Engineering, Ondokuz Mayis University, 55139 Samsun, Türkiye; ufuk.kirbas@omu.edu.tr; 2 Department of Civil Engineering, Of Faculty of Technology, Karadeniz Technical University, 61830 Trabzon, Türkiye; eiskender@ktu.edu.tr; 3 Department of Civil Engineering, Faculty of Engineering and Architecture, Recep Tayyip Erdogan University, 53100 Rize, Türkiye; tuba.kutuk@erdogan.edu.tr; 4 Graduate School of Education, Ondokuz Mayis University, 55139 Samsun, Türkiye; hayat.faridullah@gmail.com; 5 Department of Marine Engineering, Faculty of Turgut Kiran Maritime, Recep Tayyip Erdogan University, 53900 Rize, Türkiye

**Keywords:** waste, PVC foil, additive geometry, multi-scale assessment, semi-circular bending, microstructural analysis, sustainable pavement

## Abstract

While the utilization of waste polymers in asphalt mixtures is widely studied, the specific influence of additive geometry on performance mechanisms remains underexplored. This study presents a multi-scale performance assessment of asphalt mixtures modified with waste Polyvinyl Chloride (PVC) foils. Waste PVC foils were processed into two distinct geometries, “Wiry” and “Random”, and incorporated into mixture at dosages ranging from 5% to 12.5% by weight of bitumen via the dry process. At the macro-scale, Semi-Circular Bending, Hamburg Wheel Tracking, Repeated Creep, and Modified Lottman tests were conducted. At the micro-scale, Scanning Electron Microscopy and EDS analyses were employed to investigate interfacial adhesion. The results demonstrated that the “Wiry” geometry significantly outperformed the “Random” by establishing a three-dimensional reinforcement network. Specifically, the mixture modified with 7.5% “Wiry” PVC yielded the highest Flexibility Index of 24.17, representing a 3.7-fold improvement. Furthermore, this optimum dosage enhanced high-temperature stability and maintained moisture resistance (TSR > 85%), whereas dosages exceeding 10% caused agglomeration and performance loss. Microstructural imaging indicated that the fibrous morphology and calcite-rich surface of the “Wiry” additive facilitate superior mechanical interlocking. Consequently, this study suggests that optimizing waste PVC geometry is as critical as dosage for maximizing the durability and sustainability of flexible pavements.

## 1. Introduction

While rapid industrialization and urbanization rates have rendered global plastic waste management a critical environmental challenge, they have simultaneously impelled the pavement industry to seek sustainable and economic alternatives. Although the resistance of polymeric wastes to biodegradation poses a significant environmental threat, the utilization of these materials as modifiers or aggregate substitutes in hot mix asphalt (HMA) presents a significant opportunity within the framework of ‘circular economy’ principles [1]. Polymer modification has long been established as an effective strategy to enhance the performance of asphalt mixtures, particularly against prevalent pavement distresses such as rutting, fatigue, and low-temperature cracking [2]. In this context, the integration of thermoplastics, such as waste PVC, into asphalt technology holds the potential to simultaneously mitigate the waste burden and extend the service life of pavement structures.

PVC-based furniture foils, primarily used for coating wood-based panels, represent a versatile class of flexible plastic waste with significant potential for asphalt modification. These foils consist of a complex thermoplastic formulation, typically including plasticizers (15–50%), flame retardants, and stabilizers to enhance flexibility and thermal resistance [3,4]. Physically, these foils possess a density of 1.30–1.55 g/cm^3^ and a glass transition temperature between 70 and 90 °C [5]. Although their multi-component nature and surface coatings (e.g., acrylic or polyurethane lacquers) make high-grade recycling difficult, their high durability and stiffness suggest they can be effectively ‘downcycled’ as reinforcing fillers in bituminous mixtures [6]. In the context of pavement engineering, the inclusion of such flexible PVC waste offers a dual benefit: mitigating environmental plastic accumulation while potentially improving the mechanical stability of road infrastructures.

A review of the literature indicates that two primary approaches are adopted for the incorporation of waste plastics into asphalt mixtures: the “Wet Process” and the “Dry Process” [7]. In the wet process, plastics are blended into the bitumen under high temperature and shear forces to modify the binder rheology; conversely, in the dry process, plastic wastes are introduced directly to the heated aggregate, functioning as a form of “aggregate substitute” or “mixture reinforcement” [8]. It has been reported that the high modulus of elasticity and stiffness of PVC enhance the resistance of the mixture to permanent deformation at elevated temperatures by increasing the viscosity of the asphalt binder [9]. A critical parameter within this modification process is the dosage strategy. While dosage rates are typically determined based on the weight of bitumen in studies utilizing polymers as aggregate replacements, a prevalent approach in recent studies—particularly those incorporating foil or strip-type thin plastics via the dry method—involves selecting dosages within the range of 2.5% to 15% by weight of bitumen to optimize the interaction between the binder and the polymer [10,11,12,13].

Just as with the chemical structure of waste plastic, the physical form (geometry and morphology) in which it is incorporated into the mixture has a decisive impact on mechanical performance. In the literature, waste materials are typically utilized in the form of granules (pellets), powders, or fibers/strips. It is established that granular PVC enhances stiffness and Marshall stability by filling the void structure of the mixture [14]. However, it has been noted that granular particles possess limited efficacy in arresting crack propagation and may even compromise cracking resistance by inducing brittleness in the mixture when employed at high dosages [15]. Conversely, it has been observed that plastics cut into foils or strips, characterized by a high aspect ratio, establish a three-dimensional reinforcement network within the asphalt matrix [16]. This geometric configuration is associated with the ‘crack bridging’ mechanism in the literature; it is emphasized that this form significantly enhances fracture energy compared to the granular form by facilitating stress transfer within the mixture [17].

In the recent literature, a study conducted by Salman and Abbas [18] investigated the performance of recycled PVC powders derived from construction and demolition waste (specifically door and window profiles) in HMA. The researchers modified the binder via the wet process by incorporating PVC powder at rates ranging from 2.5% to 20% by weight of bitumen, subsequently conducting Marshall stability, Cantabro loss, and indirect tensile strength tests. The study findings identified 10% PVC content as the optimum dosage; at this ratio, the mixture stability reached 11.6 kN, and Cantabro loss decreased to approximately 12%, indicating enhanced resistance to disintegration. Furthermore, it was reported that while additive rates up to 10% improved moisture susceptibility (Tensile Strength Ratio [TSR] > 80%), dosages of 15% and above caused the mixture to become excessively brittle, thereby compromising its structural integrity. The authors attributed this performance decline at higher dosages to the agglomeration of PVC particles and the consequent weakening of the binder–aggregate interaction. While such studies underscore the potential of PVC as a chemical modifier, the question of whether the material can provide a physical reinforcement effect when utilized in “strip or foil” form rather than “powder”, and how distinct cutting geometries might alter this performance, remains an important research gap.

Although there is a general consensus in existing studies that waste PVC-modified asphalts exhibit increased rutting resistance, results concerning fracture resistance and moisture susceptibility show considerable variability depending on the additive geometry [19]. Despite the extensive chemical characterization of PVC in polymers, a critical research gap remains regarding how the physical morphology—specifically, the geometric cut shapes—of waste PVC foils affects the mechanical interlocking and bridging mechanisms within asphalt mixtures. To address this gap, this study investigates two distinct geometries of waste PVC foils (Wiry and Random) and evaluates their influence on mixture performance through a multi-scale framework combining mechanical performance tests and microstructural analyses.

To address this gap, this study utilizes a multi-scale performance assessment framework. A suite of advanced laboratory tests was specifically selected to simulate primary field distress mechanisms: The Hamburg Wheel Tracking Test (HWTT) was employed to simultaneously evaluate rutting resistance and moisture-induced stripping under saturated conditions, while the Repeated Creep test was selected to characterize the viscoelastic deformation and cumulative strain under repetitive loading. Furthermore, the Semi-Circular Bending (SCB) and Modified Lottman tests were integrated to determine the fracture toughness and long-term durability against moisture, respectively. To elucidate the underlying mechanisms governing these macro-scale results—such as interfacial adhesion and mechanical interlocking—micro-scale characterization techniques, including SEM, EDS, and XRD, were utilized as essential validation tools. This integrated approach aims to reveal the specific roles of ‘Wiry’ and ‘Random’ waste PVC geometries on the structural integrity and durability of asphalt mixtures.

## 2. Materials and Methods

**Figure 1:**
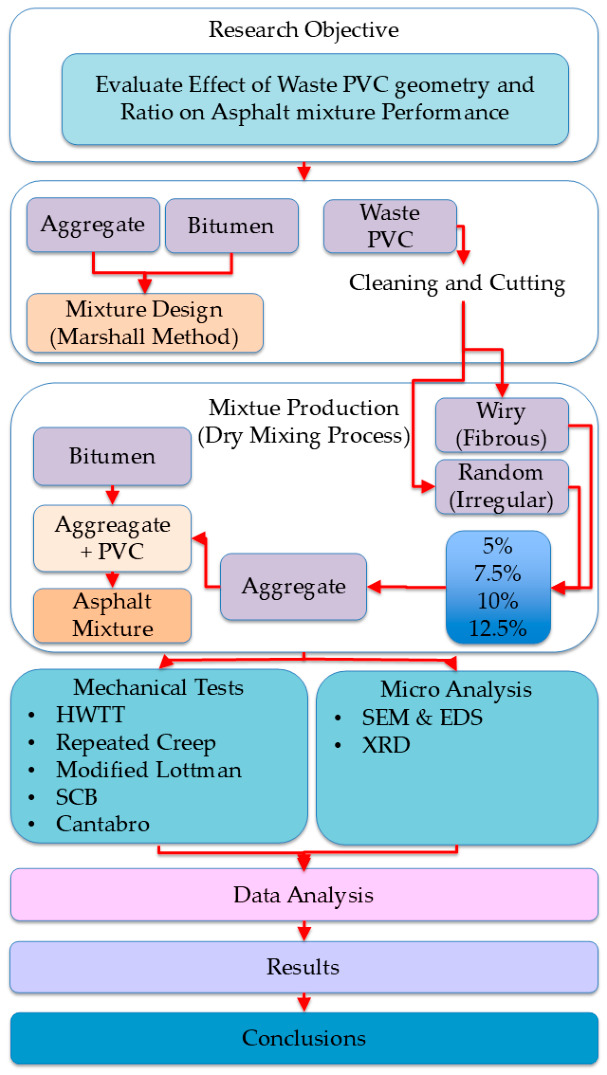
Experimental flowchart for PVC-modified asphalt mixtures.

The experimental procedure followed in this study is illustrated in Figure 1. The flowchart shows the main steps, including material selection, preparation of waste PVC in different geometries (wiry and random), mixture design, and production using the dry process, followed by testing and evaluation to determine the effects of PVC geometry and dosage on asphalt mixture performance.

### 2.1. Aggregate and Bitumen

**Figure 2:**
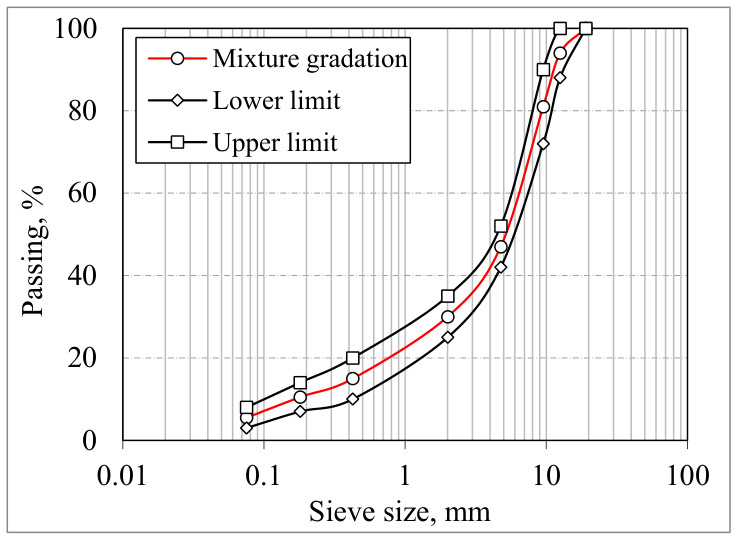
Aggregate distribution on the gradation chart.

The mineral aggregates utilized in this study were sourced from a basalt quarry located in Samsun, Türkiye. Table 1 provides the fundamental physical characteristics of the basalt aggregate, while the particle size distribution is illustrated in Figure 2. The gradation curve was developed in accordance with the “Type 1 Dense-Graded Asphalt Concrete” specification outlined in the 2023 Turkish Highway Technical Standards [20]. The aggregate composition consists of 53% coarse (No. 4 and above), 41.5% fine (No. 4–No. 200) and 5.5% filler (No. 200 and below) fractions. For binder selection, a bitumen with a penetration grade of 50/70—supplied by TÜPRAŞ Kırıkkale Refinery, Kırıkkale, Türkiye—was employed in mixture production. The key engineering properties of the binder are summarized in Table 2.

### 2.2. Preparation and Characterization of Waste PVC Additives

The waste PVC foils used in this study were originally manufactured by Plasko Plastik San. ve Tic. A.Ş. (Istanbul, Türkiye) and subsequently utilized in the furniture coating industry located in the Samsun Organized Industrial Zone. Following their use in panel coating processes, the residual PVC foils were accumulated as production waste and stored in open-air stockpiles for approximately 1 year. These post-industrial residues were collected and used as the raw material in this study, representing a post-production waste stream.

In terms of typology, the selected PVC differs from conventional waste forms (e.g., rigid profiles or granular residues) as it consists of thin, flexible, plasticized foils with surface coatings. This type was selected due to its suitability for mechanical processing into high aspect ratio particles, enabling the formation of a reinforcing network within the asphalt mixture. In contrast, more rigid or granular PVC wastes primarily act as filler materials with limited reinforcement capability.

Due to plasticizer migration and environmental exposure during open-air storage, the material is subjected to aging effects. Despite this, the PVC foils maintain their thermoplastic structure relevant for asphalt modification. Furthermore, the complex composition and absence of a true melting behavior (thermal degradation initiating at ≈250–300 °C) limit the recyclability of such materials, supporting their classification as low-value waste suitable for downcycling applications.

After procurement, the waste PVC foils were cleaned to remove impurities and subsequently processed into different geometries to evaluate the effect of morphology on mixture performance.

**Figure 3:**
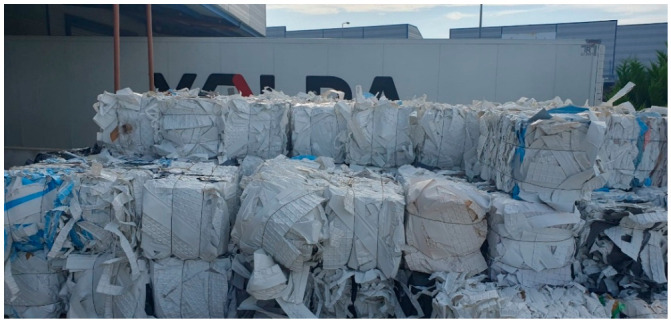
Bales of waste PVC foil collected as scrap.

**Figure 4:**
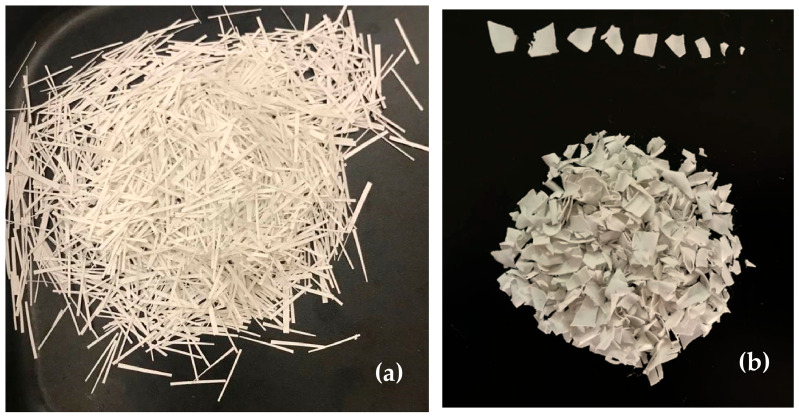
PVC foils cut into Random and Wiry shapes: (**a**) Strip-shaped (Wiry) cutting, (**b**) Random cutting.

In the first method, the waste foils were fragmented into irregular shapes and sizes to obtain the geometry designated as “Random”. To control the maximum particle size within the mixture and ensure homogeneity, the fragmented pieces were passed through a sieve with a 10 mm aperture, and the material passing through the sieve was reserved as the test sample. In the second method, precision cutting was employed to produce the “Wiry” (strip) geometry, intended to form a reinforcement network within the mixture. This process yielded strip-shaped particles with a high aspect ratio, measuring approximately 1–2 mm in width and 20 mm in length. The appearance of the raw waste PVC foils is depicted in Figure 3, while visuals of the processed “Wiry” and “Random” geometries are presented in Figure 4a and Figure 4b, respectively.

The dry process method was employed for mixture preparation. The waste PVC particles were added to the aggregates heated to 165 °C and mixed for 60 s to ensure adequate coating and prevent agglomeration. Subsequently, the bitumen was injected, and wet mixing continued for an additional 120 s. The modification method used was carried out in accordance with the dry modification method described in the literature [31]. The additive rates were determined as 5%, 7.5%, 10%, and 12.5% by weight of the optimum bitumen, based on similar studies in the literature [13] and preliminary trials. In accordance with these determined rates, modified asphalt mixtures containing varying dosages of waste PVC were prepared and subjected to performance analyses.

### 2.3. Mixture Design

The mixture design for the dense-graded asphalt concrete was conducted in accordance with the Marshall Design Method [32]. The specimens were compacted with 75 blows on each face to simulate heavy traffic conditions. The optimum bitumen content (OBC) was determined based on the target air void content of 4%. The optimum binder content determined for the control mixture (4.95%) was kept constant for both the control and PVC-modified mixtures. This approach was adopted to ensure a consistent basis for comparison of mixture performance. Adjusting the binder content separately for each modified mixture could introduce additional variability related to binder–aggregate interactions and mixture volumetrics, which might obscure the direct effect of the additive morphology. Therefore, maintaining a constant binder content allowed the observed performance differences to be attributed primarily to the presence and geometric form of the PVC additives rather than changes in binder content.

It was verified that this calculated bitumen content meets the specification criteria for other engineering properties, such as volumetric properties, stability and flow. In addition to the control mixture, eight different types of mixtures modified with waste PVC were prepared. The Marshall design test results for the control mixture, along with the specification limits set by the General Directorate of Highways (Türkiye), are presented in Table 3 and Table 4.

### 2.4. Experimental Program

#### 2.4.1. Hamburg Wheel Tracking Test

The Hamburg Wheel Tracking Test (HWTT) was conducted in accordance with AASHTO T 324 [33] to simultaneously evaluate the rutting resistance and moisture susceptibility of the asphalt mixtures. This test method is considered one of the most effective laboratory procedures for simulating the destructive effects of traffic loading under water-saturated conditions. The test was performed on cylindrical specimens submerged in a water bath maintained at a constant temperature of 50 °C. A total of four specimens per mixture were utilized. A steel wheel with a load of 705 N was passed over the specimens in a reciprocating motion.

During the test, the rut depth was recorded at specific intervals to determine the Creep Slope, Stripping Slope, and the Stripping Inflection Point (SIP), which indicates the onset of moisture damage. The test was set to terminate either after 20,000 passes or when the rut depth reached 20 mm, whichever occurred first. The specific testing parameters and operational conditions are summarized in Table 5.

#### 2.4.2. Repeated Creep Test

To evaluate the resistance of the asphalt mixtures to permanent deformation (rutting) under dynamic loading, the uniaxial cyclic compression test (Repeated Creep Test) was conducted in accordance with EN 12697-25 Method A [34]. This test simulates the traffic loads applied to the pavement structure. Cylindrical specimens were subjected to a dynamic axial stress under a cyclic loading pattern. Prior to testing, a conditioning stress was applied to ensure full contact between the loading platen and the specimen surface. Following the conditioning phase, the specimens were subjected to repeated load cycles at a controlled temperature. The cumulative permanent strain was recorded as a function of the number of load cycles. For this study, three replicates were tested for each mixture type to ensure statistical reliability. The specific operational parameters and loading conditions are summarized in Table 6.

#### 2.4.3. Modified Lottman Test (Moisture Susceptibility)

The moisture susceptibility of the asphalt mixtures was evaluated using the Modified Lottman Test in accordance with AASHTO T 283 [35]. Six cylindrical specimens were prepared for each mixture using the Marshall compaction method and were subsequently divided into unconditioned (dry) and conditioned (wet) subsets. The conditioned specimens underwent vacuum saturation to achieve 70–80% saturation, followed by a freeze cycle at −18 °C for 24 h and a thaw cycle at 60 °C for 24 h to simulate freeze–thaw damage. Prior to testing, both subsets were conditioned in a water bath at 25 °C for 2 h. The Indirect Tensile Strength (ITS) was then determined using a loading rate of 50 mm/min. The moisture resistance was quantified by the Tensile Strength Ratio (TSR), which was calculated by dividing the average ITS of the conditioned subset by that of the unconditioned subset [35]. A TSR value of 80% or higher was adopted as the threshold criterion for adequate resistance to moisture-induced damage [36].

#### 2.4.4. Intermediate Temperature Cracking Resistance (SCB Test)

To evaluate the cracking resistance of the asphalt mixtures at intermediate temperatures, the SCB Test was conducted following the Illinois Flexibility Index Test (I-FIT) protocol, in accordance with AASHTO TP124 [37,38]. This test method was selected to determine fracture mechanics-based parameters, specifically the Fracture Energy (Gf) and the Flexibility Index (FI), which have been shown to correlate strongly with field cracking performance [39,40]. Cylindrical specimens (150 mm in diameter) were used for the test. A total of six SCB replicates were tested for each asphalt group, obtained by cutting three primary compacted specimens into two equal semi-circular pieces. A vertical notch with a depth of 15 mm and a width of 1.5 mm was cut along the central axis of the flat edge to create a predetermined stress concentration zone for crack initiation. The testing was performed at a constant temperature of 25 °C using a universal testing machine (UTM) (UTEST Malzeme Test Cihazları ve Makineleri Imalat ve Dış Ticaret A.Ş. (UTEST), Ankara, Türkiye) equipped with a three-point bending fixture. The semi-circular specimens were positioned on two rollers with a support span of 120 mm. A monotonic vertical load was applied along the vertical radius of the specimen at a constant displacement rate of 50 mm/min until full fracture occurred. During the test, load and load-line displacement (LLD) data were recorded continuously. Two primary parameters were calculated: Fracture Energy (Gf), calculated by dividing the work of fracture (area under the load–displacement curve) by the ligament area (the product of ligament length and specimen thickness); and Flexibility Index (FI), calculated by dividing the Fracture Energy (Gf) by the absolute value of the post-peak slope (|m|) at the inflection point of the load–displacement curve. Higher FI values indicate a more ductile failure mode and greater resistance to crack propagation [40].

#### 2.4.5. Cantabro Test

Resistance to Abrasion and Raveling (Cantabro Test), the durability and resistance to raveling of the asphalt mixtures were evaluated using the Cantabro Loss test, in accordance with AASHTO TP 108 [41]. While originally developed for open-graded friction courses, this test is increasingly recognized as an effective method to assess the cohesion and durability of dense-graded mixtures [42]. Marshall compacted specimens (101.6 mm diameter, 63.5 mm height) were used for this test. Consistent with the other performance tests, the specimens were prepared to verify the mixture design properties. Prior to testing, the specimens were conditioned at a standard temperature of 25 °C for at least 4 h to ensure uniform thermal distribution. The initial mass of each specimen (m_initial_) was recorded with a precision of 0.1 g. The test was conducted using a standard Los Angeles (LA) abrasion machine (UTEST, Ankara, Türkiye). Unlike the aggregate toughness test, no steel spheres (abrasive charges) were placed in the drum. The cylindrical asphalt specimens were placed individually into the drum, which was then rotated at a speed of 30–33 rpm for 300 revolutions. This process simulates the abrasive action of traffic and weathering, leading to the disintegration of the specimen edges. After the completion of 300 revolutions, the specimens were carefully removed, and any loose debris was brushed off. The final mass (m_final_) was weighed. The Cantabro Loss (CL), expressed as a percentage, was calculated using Equation (1):

(1) $$CL=\frac{m_{initial}-m_{final}}{m_{initial}}\times 100$$

where CL = Cantabro Loss (%); m_initial_ = Initial mass of the specimen (g); m_final_ = Final mass of the specimen after abrasion (g). Lower CL values indicate superior resistance to raveling and stronger binder–aggregate bonding.

Although the Cantabro test was originally standardized for Open-Graded Friction Courses (OGFC) with specified loss limits of 20% for unaged and 30% for aged specimens [43], there is currently no universally mandated threshold for Dense-Graded Asphalt (DGA) mixtures. However, the recent literature regarding the durability assessment of DGA suggests that acceptable mass loss values should be lower than those permitted for open-graded mixtures. Extensive studies by Doyle and Howard [44] and Cox et al. [42] recommend that Cantabro loss values for dense-graded mixtures should ideally remain below 15% to ensure adequate resistance to raveling and general durability. Furthermore, these studies indicate that mass loss values exceeding 20% are generally indicative of poor mixture performance and insufficient cohesion.

## 3. Results and Discussion

### 3.1. Characterization of Waste PVC Additives (SEM, EDS, and XRD Analysis)

**Figure 5:**
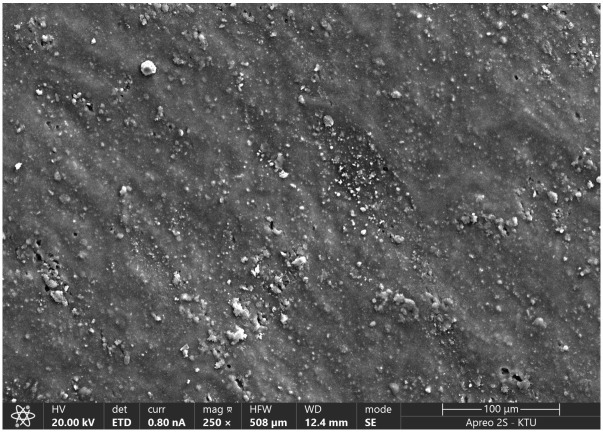

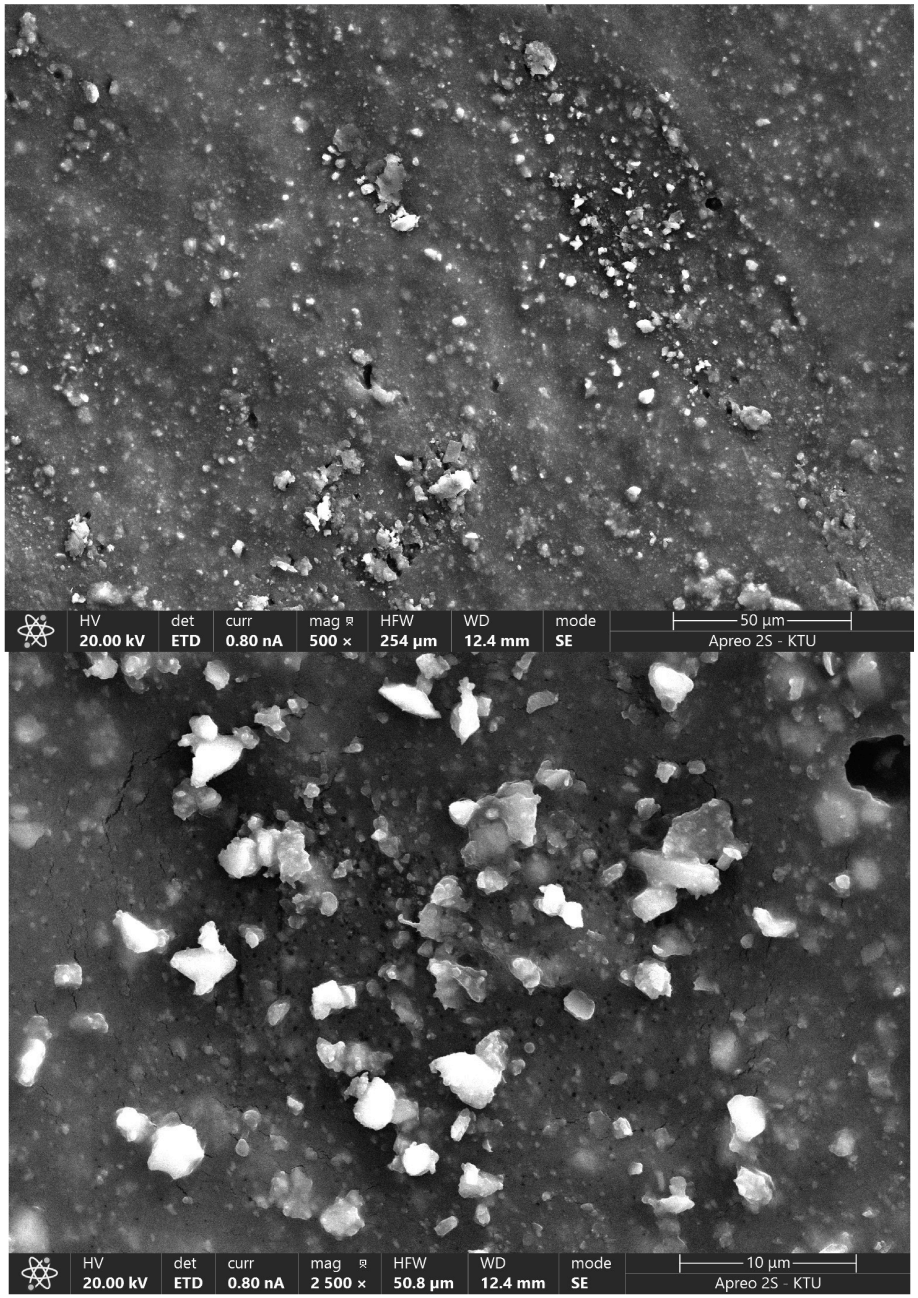

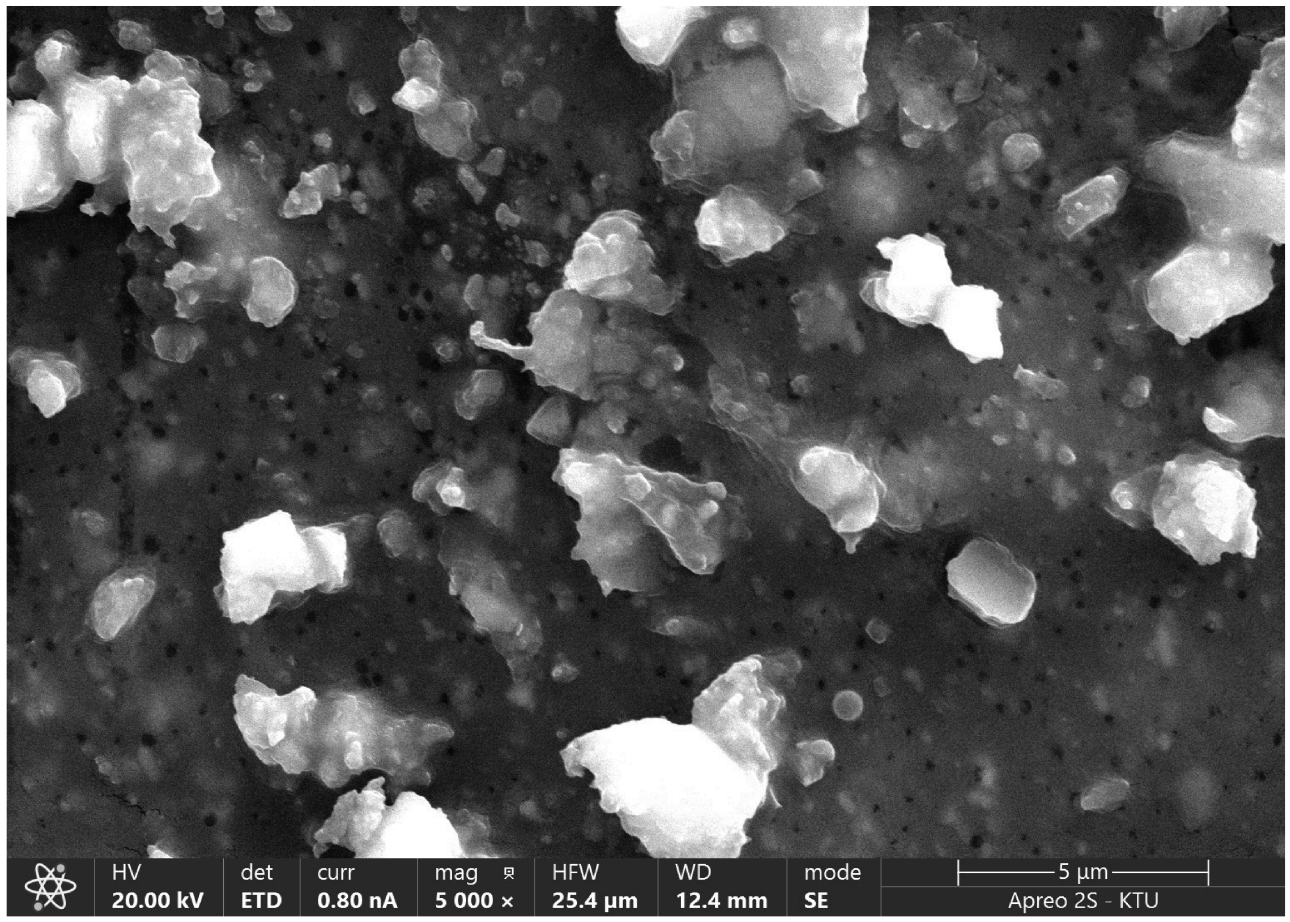
SEM micrographs of waste PVC additives.

The morphological characteristics, elemental composition, and mineralogical structure of the waste PVC foils were investigated using Scanning Electron Microscopy (SEM), Energy Dispersive X-Ray Spectroscopy (EDS), and X-Ray Diffraction (XRD). The surface microstructures of the waste additive are presented in Figure 5.

As seen in Figure 5, the waste PVC surface exhibits a highly irregular and undulated morphology characterized by directional striations. These surface features are attributed to the mechanical stresses applied during the original thermoplastic extrusion or calendering processes. A significant presence of bright phases and surface protrusions was observed, creating a rough surface texture which is critical for physical interlocking with the binder.

**Figure 6:**
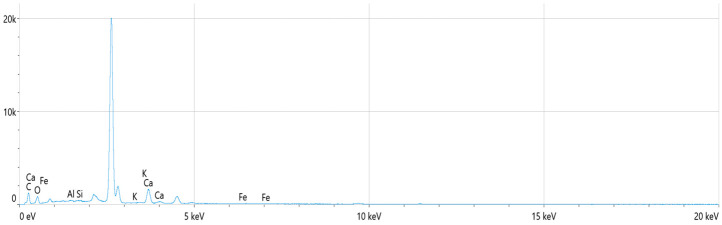
EDS spectrum.

EDS analysis was conducted to identify the chemical nature of these protrusions. The elemental composition data are summarized in Table 7 and Figure 6.

**Figure 7:**
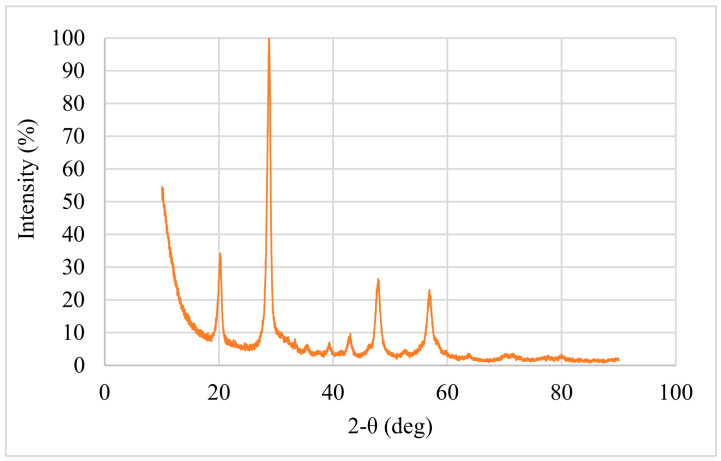
XRD pattern of waste PVC additive showing the characteristic peak of Calcite.

As shown in Table 7, the material contains a substantial mineral filler content, with Calcium (Ca) constituting approximately 26% by weight. The EDS spectrum is dominated by Carbon and Oxygen peaks, implying the polymeric matrix and carbonate structure. To definitively identify the crystalline phase of these calcium-based fillers, XRD analysis was performed. The resulting diffraction pattern is illustrated in Figure 7.

**Figure 8:**
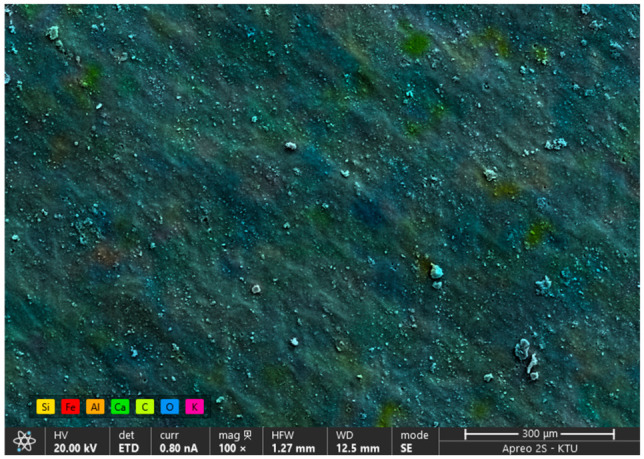
EDS elemental mapping (Si: yellow, Fe: red, Al: orange, Ca: light green, C: green, O: blue, K: pink).

**Figure 9:**
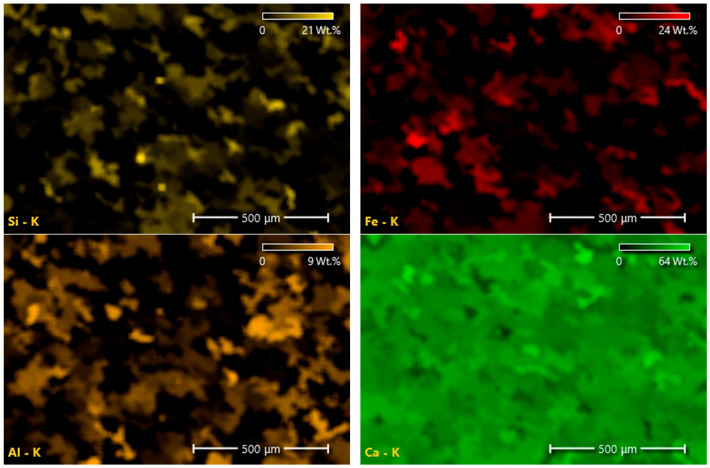

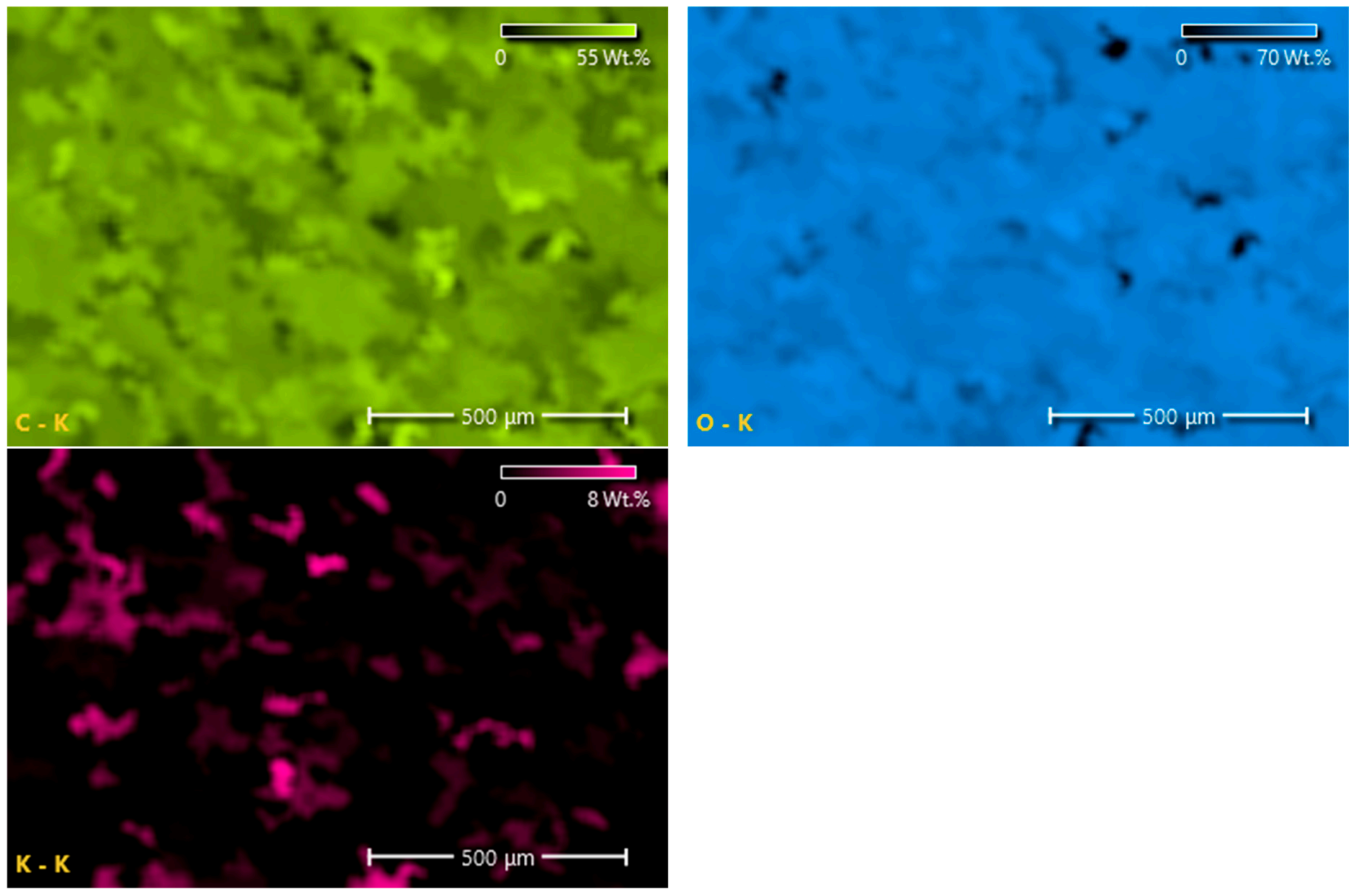
Elemental mapping.

The XRD analysis exhibits a sharp, high-intensity diffraction peak at 2-θ = 29.52°. This specific angle corresponds to the characteristic (104) plane of Calcite (CaCO_3_) [45]. This mineralogical finding corroborates the high Calcium and Oxygen concentrations observed in the EDS analysis (Figure 8 and Figure 9), implying that calcium carbonate is the primary filler material used in the PVC formulation.

The presence of rigid crystalline Calcite fillers within the polymer matrix is expected to enhance the stiffness of the waste additive, potentially contributing to the rutting resistance of the modified mixture. In addition, Calcite-containing mineral fillers are known to improve rutting resistance [46,47].

The exposed Calcite particles on the foil surface provide polar active sites. Unlike smooth, purely polymeric surfaces, these mineral sites may contribute to improved physio-mechanical interaction with the asphalt binder; however, no direct adhesion measurements were conducted to quantitatively imply this effect.

The combination of the inherently hydrophobic nature of PVC and the surface roughness induced by filler particles results in a textured surface morphology. Although such characteristics may influence wettability behavior, the potential formation of a “Cassie–Baxter” state and its effect on contact angle were not directly evaluated in this study. Therefore, any associated improvement in moisture resistance should be interpreted as a possible mechanism rather than a directly verified outcome.

### 3.2. Hamburg Wheel Tracking Test Results and Evaluation

**Figure 10:**
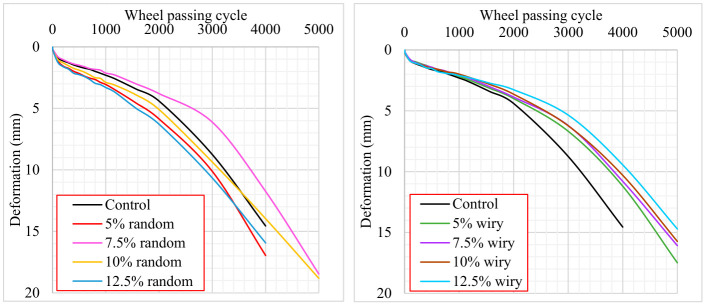
Average deformation curves of randomly and wiry-cut waste PVC modified asphalt mix samples.

The HWTT was performed in water at 50 °C. The rut progression curves plotted by averaging the deformations (left and right wheel) are given in Figure 10. Furthermore, common index parameters from HWTT results were calculated and presented in Table 8.

The HWTT was conducted in accordance with AASHTO T 324 using the wet test method. The termination threshold was set at a maximum rut depth of 20 mm or 20,000 passes. It was observed that both control and modified mixtures reached the 20 mm failure limit at approximately 5000 passes. Considering that the tests were performed submerged in water without the addition of any anti-stripping agents or secondary additives, these results are considered reasonable and representative of severe moisture-induced stress.

It should be noted that AASHTO T 324 does not prescribe a universal pass/fail criterion for the number of loading cycles, as such limits are typically defined by regional specifications. In the Turkish Highway Technical Specifications issued by the General Directorate of Highways (KGM), a rut depth limit of 4.5 mm at 20,000 passes is specified for dry Hamburg Wheel Tracking tests conducted at 60 °C using 50 mm thick specimens. However, a corresponding limit value for the wet test condition has not been explicitly defined. Therefore, in this study, the performance of the mixtures under wet HWTT conditions was evaluated based on their relative resistance to deformation and moisture-induced damage by comparing the stripping behavior and failure points of the modified mixtures with those of the control mixture.

The HWTT results suggest that the geometric configuration of waste PVC additives may influence the thermo-mechanical performance and moisture susceptibility of asphalt mixtures. When analyzing the resistance to permanent deformation through Creep Slope (CS) and total deformation values, the fibrous structure of wiry PVC particles provided a distinct reinforcement mechanism within the asphalt matrix. The mixture containing 12.5% wiry PVC exhibited superior structural stability among all tested samples, achieving the lowest CS value of 0.00137 mm/cycle and limiting total deformation to 9.44 mm, which represents a substantial improvement over the control sample’s CS of 0.002113 mm/cycle and deformation of 14.56 mm. This behavior indicates that the wiry geometry effectively distributes shear stresses and delays the accumulation of rutting through a bridging effect between aggregates.

In contrast to the consistent stability observed in the wiry series, the mixtures modified with random geometry PVC displayed considerable variability and instability in their deformation behaviors. While the 7.5% random addition yielded an optimal resistance with a reduced CS of 0.0018775 mm/cycle, deviations from this specific ratio resulted in marked performance deterioration. Notably, the deformation curves for the 12.5% random mixture reveal a rapid descent into the stripping phase, suggesting that high concentrations of randomly cut plastics with larger surface areas may disrupt the aggregate-binder adhesion rather than reinforcing it. This is further evidenced by the 5% random sample, which performed worse than the control mixture with a total deformation of 16.96 mm, implying the sensitivity of the matrix to the random additive ratio.

The moisture susceptibility of the mixtures was further characterized by the Stripping Inflection Point (SIP) and the Stripping Slope (SS), where a higher SIP indicates prolonged resistance to water damage [48]. The data reveals that wiry additives generally maintained or improved the stripping resistance compared to the control value of 2515 cycles, with the 5% wiry sample reaching a SIP of 3025 cycles. However, the 12.5% random mixture exhibited a critical premature failure with a SIP of only 1775 cycles, followed by a steep stripping slope that indicates a rapid loss of adhesion under hydrodynamic pressure. Consequently, the comprehensive analysis suggests that wiry geometries are preferable for enhancing the internal cohesion and service life of the pavement, whereas random geometries pose a risk of accelerated moisture-induced damage if not utilized at the precise optimum content.

### 3.3. Repeated Creep Test Results and Evaluation

**Figure 11:**
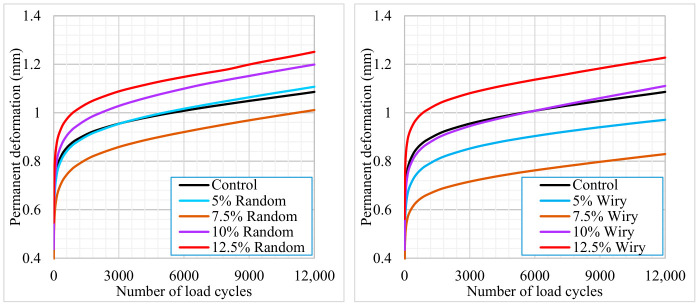
Repeated creep test results.

A Repeated Load Creep Test was conducted to evaluate the permanent deformation resistance of asphalt mixtures modified with waste PVC coating materials of varying geometries (fibrous and random). The experiments were performed at a test temperature of 40 °C. To ensure the accuracy and reproducibility of the results, three identical asphalt specimens were tested for each additive configuration. The load repetition–deformation curves presented in Figure 11 were plotted using the arithmetic mean of the data obtained from these three replicate specimens.

Upon examining the Repeated Load Creep Test results presented in Figure 11, it is evident that the geometry and content of waste PVC significantly influence the permanent deformation behavior.

For both waste geometries (wiry and random), the lowest permanent deformation values were obtained at the 7.5% additive content. At lower dosages (5% and 7.5%), the reinforcement effect of the fibrous (wiry) structure provided a significantly superior performance compared to the random structure. Specifically, at the 7.5% content, wiry samples exhibited approximately 22% lower total deformation than random samples, indicating enhanced shear resistance. Similarly, the wiry geometry offered a 14% performance advantage at the 5% content.

One of the most notable findings of the analysis is the reversal of performance when the additive content exceeds 10% (up to 12.5%). The graphs reveal that mixtures with 10% and 12.5% contents (both wiry and random) exhibited higher deformation values compared to the Control (unmodified) specimen. This suggests that excessive plastic volume within the matrix weakens aggregate interlocking, effectively creating slip planes that accelerate deformation.

The creep slopes calculated from the secondary (linear) region of the deformation curves reflect the stability of the mixtures under long-term loading. Consistent with the total deformation results, the lowest creep slope values were calculated for the 7.5% Wiry (0.000014 mm/cycles) and 5% Wiry (0.000015 mm/cycles) specimens. A lower creep slope values are generally associated with greater resistance to viscoplastic behavior. Conversely, the increase in slopes when deviating from the optimum content implies the mixture’s increased susceptibility to rutting.

### 3.4. Modified Lottman Test Results and Evaluation

**Figure 12:**
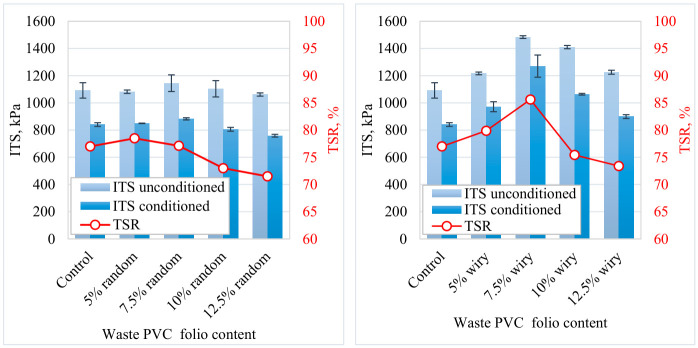
Influence of PVC dosage on ITS and TSR values.

Figure 12 illustrates the effects of different waste PVC foil geometries (random and wiry) and varying additive contents on the Indirect Tensile Strength (ITS) and Tensile Strength Ratio (TSR) of the asphalt mixtures. The obtained data reveal that both fiber geometry and dosage are decisive parameters influencing mechanical performance and moisture resistance.

A comparative analysis of Figure 12 shows that wiry-shaped waste PVC additives are more effective in enhancing the mixture’s resistance to tensile stresses compared to random-shaped additives. Due to their low aspect ratio and irregular structure, random geometry particles failed to create a sufficient reinforcement effect within the aggregate skeleton; consequently, their ITS values followed a horizontal trend similar to the control sample levels (approximately 1100 kPa). Conversely, wiry fibers, by virtue of their morphology, facilitated stronger mechanical interlocking between the aggregate and the bitumen matrix. This mechanism hinders the pull-out of fibers under loading, allowing tensile stresses to be dissipated over the fibers and thereby increasing overall strength.

For both series, the 7.5% additive rate emerges as a critical threshold regarding performance evolution. Particularly in the wiry fiber series, ITS values exhibited a linear escalation as the content increased from 0% to 7.5%, reaching a peak point at approximately 1500 kPa. This improvement can be attributed to the enhancement of binder cohesion via polymer modification and the crack-arresting effect of the fibers.

However, as the additive content exceeded the optimum value, reaching 10% and 12.5%, a precipitous decline in mechanical performance was observed in both series. Consistent with the existing literature, this phenomenon is primarily caused by the inability of high plastic content to distribute homogeneously within the mixture, leading to agglomeration. These plastic clusters create weak spots that are not fully wetted by bitumen, disrupting the structural continuity of the mixture and reducing its load-bearing capacity.

When examining the TSR results, which characterize the mixture’s durability against moisture, it is observed that the 7.5% wiry PVC addition improved the bitumen–aggregate interfacial adhesion, elevating the TSR value to the 85% level (surpassing the control sample). The use of hydrophobic PVC material at this optimum ratio effectively limited water penetration into the mixture body. However, increasing the dosage further (10–12.5%) reduced the coating ratio of the bitumen film over the aggregates, facilitating water ingress at the interface and causing stripping. This is corroborated by the fact that the TSR values of high-dosage samples dropped below the critical 75% threshold.

In conclusion, the utilization of waste PVC foils with a “wiry” geometry at a limiting dosage of 7.5% has been determined as the optimal engineering solution for maximizing dry strength (ITS) while simultaneously maintaining resistance to water damage (TSR).

### 3.5. Semi-Circular Bending Test Results and Evaluation

**Figure 13:**
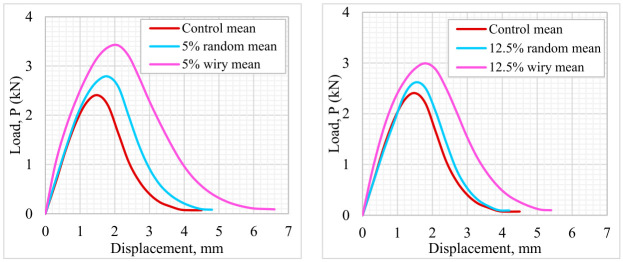

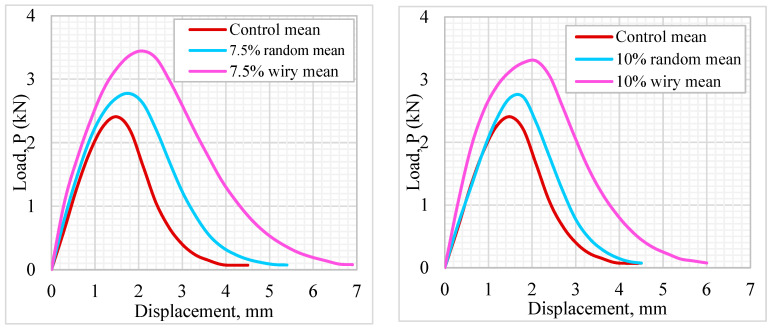
Load–displacement curves of mixtures modified with random and wiry-shaped PVC.

**Figure 14:**
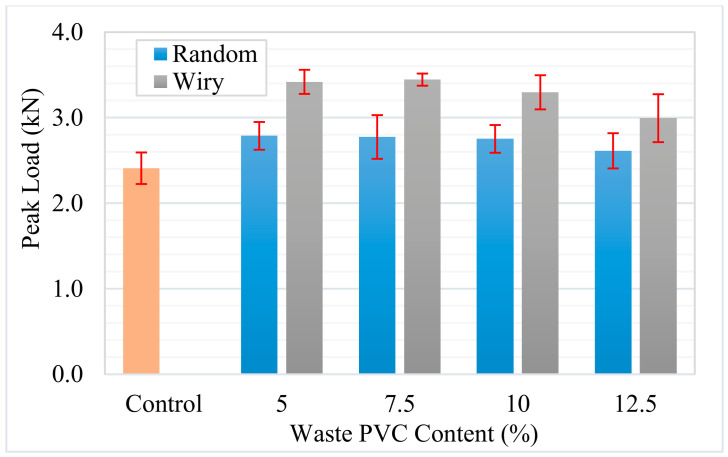
Effect of waste PVC content and geometry on peak load.

The SCB test was conducted to evaluate the fracture resistance of the asphalt mixtures. Load–displacement curves of the asphalt mixture samples are shown in Figure 13. The peak load values and standard deviations obtained from these tests are presented in Figure 14. The results indicate that it is evident that the incorporation of waste PVC is associated with an improvement in the load-bearing performance of the samples compared to the control mixture. From a geometric perspective, the “Wiry” type additive appears to provide more effective interlocking mechanism within the matrix compared to the “Random” type, resulting in higher strength values. The data indicate that the optimum waste PVC content is approximately 7.5%. Beyond this ratio—specifically, at 10% and 12.5% contents—a loss in strength was observed. This decrease may be related to the weakening of binder–aggregate adhesion and/or the tendency of PVC particles to agglomerate at elevated dosages.

**Figure 15:**
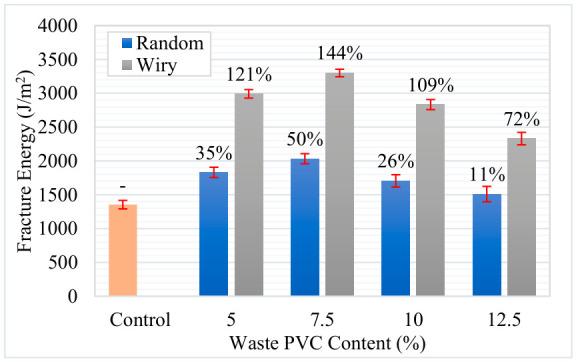
Effect of waste PVC content and geometry on fracture energy.

Using the load–displacement curves obtained from the SCB tests, the fracture energy (Gf) was calculated, serving as a critical parameter to define the asphalt concrete’s resistance (toughness) against crack propagation. As illustrated in Figure 15, the use of waste PVC increased the fracture energy in all modified mixtures compared to the control sample, which had a value of 1355 J/m^2^. This improvement suggests that waste PVC particles enhance the energy absorption capacity by impeding crack propagation or extending the crack path within the matrix. The impact of particle geometry was significant; the performance of “Wiry” (fibrous) waste was markedly superior to that of “Random” particles. For instance, at a 7.5% addition rate, while the “Random” series provided a 50% increase over the control (2033 J/m^2^), the “Wiry” series at the same rate achieved a 144% increase, reaching the highest observed value of 3301 J/m^2^. This disparity is explained by the effective bridging mechanism formed by the fibrous PVC structure across crack surfaces, which significantly enhances the ductility of the specimen.

**Figure 16:**
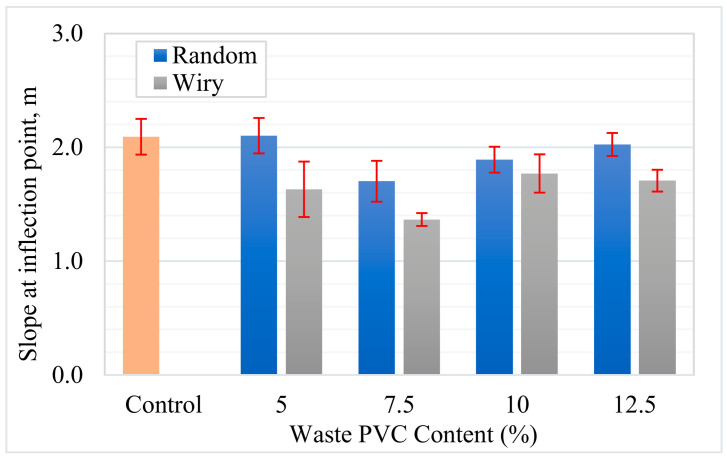
Crack propagation of asphalt mixtures.

To further characterize the cracking potential, the slope at the inflection point (|m|) and the Flexibility Index (FI) were analyzed. Figure 16 presents the variations in slope values. The results indicate that waste additives affect the cracking resistance of asphalt mixtures, depending on the geometry and content rate. The reference control sample exhibited a brittle behavior with an FI value of 6.47. In the literature, FI values below 10 are generally regarded as indicators of high brittleness and weak resistance to crack propagation [37,40]. Conversely, both additive types improved the mixture’s energy damping capacity. The performance of the “Wiry” additive was particularly notable; at 7.5% content, it not only maximized fracture energy but also reduced the absolute slope (|m|) from 2.093 to 1.366. This reduction appears to indicate that the material continues to carry load rather than experiencing sudden failure at the moment of fracture, thereby acquiring ductile behavior.

**Figure 17:**
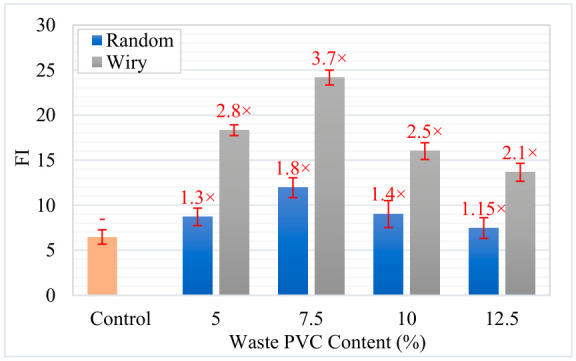
Flexibility index of waste PVC modified asphalt mixtures.

Consequently, the FI values, summarized in Figure 17, highlight the superior performance of the 7.5% Wiry mixture. This specific mixture achieved an FI of 24.17, representing an approximate 3.7-fold improvement over the control sample. This mechanism of increase is described in the literature as the “crack bridging” effect, where fibrous structures connect micro-cracks within the matrix, transferring stresses and delaying crack progression [1,16]. While “Random” geometry additives also enhanced performance, reaching a maximum FI of 11.94 at the 7.5% rate, they were not as effective as the “Wiry” type. This difference is attributed to the “Wiry” structure providing better interlocking with the aggregate matrix and more efficient load transfer. For both additive groups, 7.5% was identified as the optimum usage amount. When the additive rate was increased to 10% and 12.5%, a decline in FI values was observed; for example, the FI for 12.5% Wiry decreased to 13.65. This loss of performance at high rates is associated with the agglomeration of additives within the mixture and insufficient coating of aggregates by bitumen, leading to the formation of weak adherence zones [49]. Similar studies have reported that excessive fiber/additive usage reduces workability and negatively affects air void distribution, thereby degrading mechanical performance [50]. In conclusion, the use of 7.5% “Wiry” waste additive stands out as the most suitable modification to maximize the intermediate temperature cracking resistance of asphalt mixtures.

### 3.6. Cantabro Test Results and Evaluation

**Figure 18:**
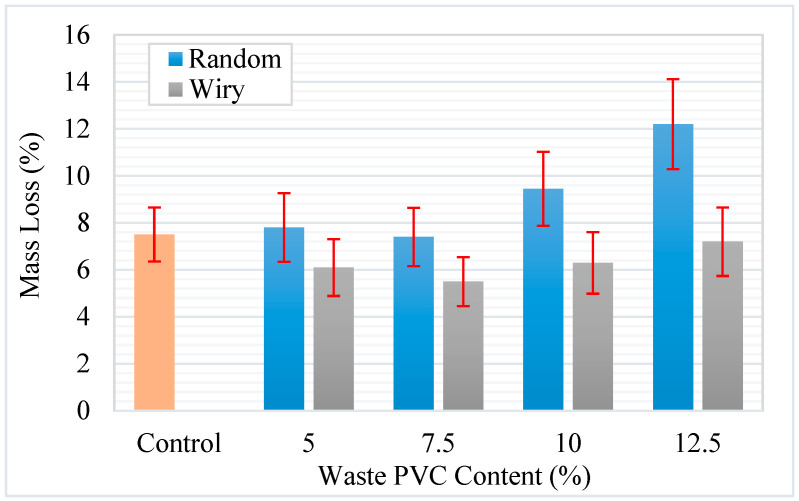
Cantabro mass loss of waste PVC modified asphalt mixtures.

The Cantabro loss test was conducted to evaluate the resistance of the asphalt mixtures to disintegration and raveling under abrasive forces. The percentage of mass loss for the control and modified mixtures is presented in Figure 18.

The results indicate a distinct divergence in performance governed by the geometric shape of the waste PVC additives. The control mixture exhibited a mass loss of approximately 7.6%. Upon analysis of the “Wiry” series, it is observed that the inclusion of fibrous PVC particles improved the durability of the mixture, reducing the mass loss to a minimum of 5.6% at the optimum content of 7.5%. This improvement is attributed to the reinforcement mechanism provided by the wire-like structures, which act as a three-dimensional network within the matrix, holding the aggregate particles together and preventing dislodgement during the tumbling process [51]. Conversely, the “Random” series demonstrated a negative trend; as the content of random-shaped PVC increased, the mass loss rose significantly, reaching approximately 12.2% at the 12.5% substitution rate. This deterioration suggests that the non-fibrous, random particles may disrupt the interlocking of the aggregate skeleton and weaken the bitumen–aggregate adhesion, thereby making the mixture more susceptible to raveling [42]. While the “Wiry” modification enhanced the resistance to abrasion, the excessive mass loss observed in high-content “Random” samples highlights the critical importance of additive geometry in maintaining the structural integrity of the asphalt surface course.

It should be noted that, although the Cantabro test is widely used for evaluating raveling resistance, its application to dense-graded asphalt mixtures remains limited, and no universally accepted specification limits are available for such mixtures. Previous studies have indicated that the Cantabro test can be used as a relative indicator of mixture durability rather than an absolute performance criterion. It is reported that mass loss values for dense-graded asphalt mixtures were generally below 15%, highlighting the potential of the test as a comparative evaluation tool rather than a specification-based acceptance method [44]. In addition, the Cantabro test can also be effectively used to evaluate the mixing efficiency and dispersion quality of modifiers within dense-graded asphalt mixtures [52].

In this context, the Cantabro results obtained in the present study (ranging approximately between 5% and 12%, as shown in Figure 18) are consistent with the ranges reported in the literature for dense-graded mixtures. Therefore, the performance of the mixtures was assessed on a comparative basis, focusing on the relative differences between the control and PVC-modified mixtures. The results indicate that the use of wiry-shaped PVC additives leads to lower mass loss values compared to random-shaped particles, suggesting improved resistance to raveling. This behavior may also be associated with a more effective distribution and interaction of the wiry PVC within the mixture structure. However, these findings should be interpreted within the framework of relative performance evaluation, given the absence of standardized threshold values for dense-graded mixtures.

### 3.7. Statistical Evaluation of Experimental Results

Two-way analysis of variance (ANOVA) was performed to evaluate the effects of PVC geometry and additive content on the performance of asphalt mixtures. The results are summarized in Table 9.

The analysis showed that both geometry and dosage had statistically significant effects on most performance parameters, including Cantabro loss, TSR, and FI (*p* < 0.05). Geometry appears to play a dominant role in rutting resistance (HWTT) and cracking resistance (FI), while dosage has a more pronounced influence on permanent deformation (RCT).

A significant interaction effect between geometry and dosage was observed for TSR and FI (*p* < 0.05), indicating that moisture susceptibility and cracking behavior are influenced by the combined effect of additive morphology and content. In contrast, Cantabro loss and HWTT results were primarily affected by independent contributions of these factors.

Following the ANOVA, post hoc comparisons were conducted to further examine the differences between mixture groups across all evaluated performance parameters. The results indicated that mixtures modified with wiry-shaped PVC generally exhibited superior performance compared to both control and random-modified mixtures, particularly for FI and TSR. Differences among mixture groups for Cantabro loss, HWTT, and RCT parameters were comparatively less pronounced.

These findings suggest that different performance mechanisms respond differently to PVC modification. Overall, the statistical analysis supports the experimental observations and indicates that optimum performance is observed at moderate additive contents, particularly for wiry-shaped PVC.

The experimental results identifying an optimum PVC content of 7.5% demonstrate a high degree of correlation with theoretical thresholds and practical benchmarks established in the literature. Previous studies on polymer-modified asphalt mixtures have indicated that plastic contents in the range of approximately 5–10% by weight of the binder provide an optimal balance between mechanical performance and workability [7,19]. Beyond this critical concentration, the excessive increase in kinematic viscosity often impedes the workability and compactability of the mixture, while simultaneously predisposing the pavement to low-temperature thermal cracking. Therefore, the 7.5% dosage achieved in this study represents an ideal equilibrium between maximizing the mechanical properties of asphalt mixtures.

Furthermore, the superior performance of the “wiry” (fibrous/slender) cutting geometry compared to “random” (granular) distribution can be elucidated through the reinforcement effect theory in composite materials. The elongated morphology of wiry PVC particles functions as a micro-reinforcing network within the aggregate–bitumen matrix, enhancing load transfer mechanisms and mitigating tensile stresses. A previous study [53] suggests that a higher aspect ratio in polymer additives strengthens the interfacial adhesion between the binder and the aggregate, thereby increasing the cohesive resistance of the asphalt concrete. This morphological advantage explains the enhanced stiffness modulus and fatigue life observed in samples modified with wiry-cut waste.

The HWTT results indicate that the mixture containing 12.5% wiry PVC exhibited the lowest deformation and creep slope values, suggesting superior resistance to permanent deformation under water-submerged, high-temperature conditions. However, this observation differs from the outcomes of the other performance tests, where the optimum dosage was consistently identified as 7.5%. This discrepancy may be attributed to the intrinsic characteristics of the HWTT, which is performed under submerged conditions and simultaneously captures rutting and moisture-induced damage. The evolution of deformation in this test is governed not only by shear resistance, but also by changes in adhesion and cohesion within the mixture. While higher PVC contents may improve resistance to deformation through increased stiffness, they may also alter the binder–aggregate interaction and internal cohesion. Therefore, the coupled influence of moisture susceptibility and rutting behavior in the HWTT may lead to performance trends that differ from those observed in tests where these mechanisms are evaluated independently.

A general decline in the mechanical performance of asphalt mixtures was observed at higher PVC contents (10% and 12.5%). This deterioration may be attributed to the agglomeration of PVC particles at elevated dosages, which can negatively affect mixture homogeneity and internal structure. This behavior is further supported by the consistent deterioration observed in TSR, Cantabro loss, and creep performance at higher dosages. These combined trends indicate a loss of mixture homogeneity and the formation of weak zones within the asphalt matrix, which may be associated with particle agglomeration.

Although this study primarily focuses on the mechanical performance of waste PVC-modified asphalt mixtures, the use of waste-derived materials also presents potential environmental benefits. The incorporation of waste PVC foils can contribute to reducing plastic waste accumulation and support circular economy principles by diverting materials from landfills into infrastructure applications. In addition, partial replacement of conventional materials with waste-based additives may reduce the demand for virgin resources. However, the environmental implications of this approach were not quantitatively evaluated in this study; therefore, these benefits should be interpreted qualitatively. Potential limitations, including processing-related energy consumption and long-term environmental considerations, should also be acknowledged.

In addition to sustainability aspects, practical considerations such as workability, mixing uniformity, and field applicability should also be taken into account. The incorporation of waste PVC additives through the dry process may influence mixture handling and compaction behavior, depending on particle shape and content. In this study, the adopted mixing protocol provided a relatively homogeneous distribution of the additive, particularly for wiry-shaped PVC, which appeared to enhance interaction within the mixture structure. However, the potential for agglomeration, especially at higher additive contents or with irregular particle geometries, should be considered. From a practical perspective, the use of waste PVC is not expected to require significant modifications to conventional asphalt production processes; however, careful control of mixing conditions may be necessary to ensure uniform dispersion. Further investigation under plant and field conditions is recommended to confirm constructability and long-term performance.

## 4. Conclusions

This study presented a multi-scale experimental investigation to evaluate the effects of waste PVC foil geometry (“Wiry” vs. “Random”) and content on the performance of asphalt mixtures. Based on micro-scale analyses (SEM/EDS/XRD) and macro-scale tests, the following conclusions were drawn:

- Microstructural analyses revealed that the “Wiry” (fibrous) morphology facilitated a superior mechanical interlocking within the aggregate matrix compared to the “Random” geometry. Furthermore, the presence of calcite fillers within the PVC structure provided a rougher surface texture, enhancing the physico-chemical adhesion potential between the polymer and the bitumen.
- Waste PVC improved high-temperature performance. The “Wiry” geometry showed a stronger reinforcement effect, reducing deformation more effectively, while the “Random” geometry exhibited instability at high dosages.
- The 7.5% “Wiry” mixture achieved the highest Flexibility Index (FI = 24.17), indicating a transition toward more ductile behavior. Although “Random” PVC improved fracture energy, its effectiveness remained limited.
- Durability was strongly dependent on geometry. “Wiry” additives reduced Cantabro mass loss and maintained TSR values above 85% at optimum content, whereas higher dosages (>10%) negatively affected performance.
- The optimum PVC content was identified as 7.5% by weight of the binder. Higher contents led to performance deterioration due to agglomeration and reduced mixture homogeneity.
- In conclusion, waste PVC foils can be effectively used as a sustainable modifier, provided that a wiry (fiber-like) geometry is used and the dosage is limited to the optimum level (7.5%). Further studies should focus on field validation under real conditions.
- From a practical implementation perspective, the proposed dry-process incorporation of waste PVC can be applied within conventional asphalt production systems without requiring major modifications to plant equipment. However, controlling mixing conditions is essential to ensure uniform dispersion and to prevent agglomeration.

## 5. Recommendations and Limitations

Based on the findings of this study, the use of waste PVC foils in asphalt mixtures appears to be a promising modification approach, particularly when applied in a wiry (fiber-like) geometry. The results suggest that an additive content of approximately 7.5% by weight of the binder may provide a balanced improvement in mechanical performance. The adopted mixing protocol (30 s dry mixing followed by 90 s wet mixing at approximately 165 °C) was observed to produce a relatively homogeneous distribution of the additive and reduce agglomeration under the conditions of this study.

Despite these findings, several limitations should be acknowledged. The selected mixing procedure was based on literature and laboratory experience and was not systematically optimized; therefore, variations in mixing time, temperature, or sequence may influence additive dispersion and overall performance. In addition, the binder content (4.95%) was kept constant for all mixtures to enable direct comparison, although mixture-specific optimization could lead to different performance outcomes. Furthermore, the absence of binder-level rheological characterization (e.g., DSR or MSCR tests) limits a detailed interpretation of the modification mechanisms at the binder scale. Finally, the results are based on laboratory-scale testing, and further validation under field conditions is required to confirm long-term performance and practical applicability.

## Figures and Tables

**Table 1:** Physical properties of the aggregate.

Test	Test Standard	Test Result	Specification Limit [20]
Bulk specific gravity (coarse agg.)	ASTM C127 [21]	2.566	-
Apparent specific gravity (coarse agg.)	ASTM C127	2.580	-
Bulk specific gravity (fine agg.)	ASTM C128 [22]	2.570	-
Apparent specific gravity (fine agg.)	ASTM C128	2.713	-
Specific gravity (filler)	ASTM D854 [23]	2.58	-
Los Angeles abrasion (%)	AASHTO T96 [24]	24.1	≤27
Flakiness (%)	BS 812 [25]	12	≤25
Water absorption (Coarse agg.) (%)	ASTM C127	0.28	≤2.0
Water absorption (fine agg.) (%)	ASTM C127	1.93	≤2.0

**Table 2:** The results of tests conducted on the 50–70 penetration asphalt binder.

Test	Standard	Result
Specific gravity at 25 °C (g/cm^3^)	ASTM D70 [26]	1.022
Softening point (°C)	ASTM D36 [27]	50.5
Flash point (°C)	ASTM D92 [28]	264
Penetration (100 g, 5 s, 25 °C), 0.1 mm	ASTM D5 [29]	52.1
Ductility (25 °C, 5 cm/min), cm	ASTM D113 [30]	>150

**Table 3:** Volumetric properties of control and wiry-PVC modified asphalt mixtures.

Design Parameter	Mixture Type					Board in Türkiye [20]	
	Control	5% Wiry	7.5% Wiry	10% Wiry	12.5% Wiry	Min.	Max.
Compaction, blows	75	75	75	75	75	75	75
Bulk specific gravity, G_mb_	2.474	2.472	2.469	2.465	2.463	-	-
Air voids, V_a_, %	4.01	4.19	4.21	4.37	4.45	3	5
Voids in mineral aggregate, VMA, %	14.1	14.19	14.29	14.43	14.50	14	16
Void filled with asphalt, V_f_, %	71.6	71.1	70.5	69.7	69.3	65	75
Marshall stability, kg	1175	1251	1488	1464	1368	900	-
Flow, F, mm	3.01	3.22	3.26	3.35	3.43	2	4

**Table 4:** Volumetric properties of random-PVC modified asphalt mixtures.

	Mixture Type				Board in Türkiye [20]	
Design Parameter	5% Random	7.5% Random	10% Random	12.5% Random	Min.	Max.
Compaction, blows	75	75	75	75	75	75
Bulk specific gravity, G_mb_	2.471	2.470	2.469	2.462	-	-
Air voids, V_a_, %	4.15	4.16	4.21	4.48	3	5
Voids in mineral aggregate, VMA, %	14.24	14.25	14.29	14.54	14	16
Void filled with asphalt, V_f_, %	70.8	70.8	70.5	69.2	65	75
Marshall stability, kg	1218	1336	1374	1287	900	-
Flow, F, mm	3.15	3.21	3.18	3.10	2	4

**Table 5:** Hamburg wheel tracking test parameters.

Test Parameters	Values
Standard Designation	AASHTO T 324
Test Temperature	50 °C
Load Type	Steel Wheel (705 ± 4.5 N)
Maximum rut depth before termination	20 mm
Tracking rate	52 ± 2 passes/min (approx. 26 cycles/min)
Conditioning Time	30 min (submerged prior to testing)
Maximum passes	20,000
Time of conditioning in the water bath	30 min
Termination Criteria	20,000 passes or 20 mm rut depth
Conditioning cycles	5

**Table 6:** Repeated creep test parameters.

Test Parameters	Value/Condition
Test Temperature	40 °C
Conditioning Stress	3 kPa
Conditioning Time	2 min
Axial Test Stress	95 kPa
Loading Frequency	0.5 Hz
Number of Load Cycles	12,000

**Table 7:** Elemental composition of waste PVC determined by EDS analysis.

Element	Atomic %	Atomic % Error	Weight %	Weight % Error	Net Counts
C	32.1	0.4	21.7	0.3	7552
O	54.6	1.2	49.3	1.1	5004
Al	0.6	0.1	0.8	0.1	654
Si	1.1	0.1	1.7	0.2	1472
K	0.0	—	0.0	—	0
Ca	11.5	0.2	26.0	0.4	17,778
Fe	0.1	0.1	0.5	0.3	129

**Table 8:** Common index parameters calculated from HWTT results.

Mixture	Stripping Inflection Point (SIP) (Cycling)	Creep Slope (CS) (mm/Cycling)	Inverse Creep Slope (ICS) (Cycling/mm)	Stripping Slope (SS) (mm/Cycling)	Inverse Stripping Slope (ISS) (tur/Cycling)	Deformation at 4000-Wheel Cycling (mm)
Control	2515	0.002113	475	0.0058	172.5	14.556282
5% random	2310	0.002605	385.5	0.0066815	156	16.960516
7.5% random	3015	0.0018775	542.5	0.005771	174	11.748231
10% random	1870	0.0019035	547	0.0045485	224.5	13.964666
12.5% random	1775	0.00243	414.5	0.004871	205.5	15.938185
5% wiry	3025	0.002098	479	0.004899	204	11.194281
7.5% wiry	2900	0.001892	528.5	0.004691	227.5	10.793106
10% wiry	2350	0.001562	676.5	0.0038875	258.5	10.302968
12.5% wiry	2750	0.00137	797	0.004158	252.5	9.440007

**Table 9:** Summary of two-way ANOVA results for performance parameters.

Parameter	Factor	F-Value	*p*-Value	Significance
Cantabro Loss	Geometry	25.5	<0.001	Significant
	Dosage	6.06	0.006	Significant
	Geometry × Dosage	1.7	0.207	Not significant
TSR	Geometry	97.01	<0.001	Significant
	Dosage	90.58	<0.001	Significant
	Geometry × Dosage	39.87	<0.001	Significant
Deformation (RCT)	Geometry	3.41	0.083	Not significant
	Dosage	5.69	0.008	Significant
	Geometry × Dosage	0.34	0.799	Not significant
Creep Slope (HWTT)	Geometry	322.77	<0.001	Significant
	Dosage	2.6	0.124	Not significant
	Geometry × Dosage	3.38	0.075	Not significant
SIP (HWTT)	Geometry	6.2	0.038	Significant
	Dosage	3.49	0.07	Not significant
	Geometry × Dosage	1.27	0.348	Not significant
FI (SCB)	Geometry	403.32	<0.001	Significant
	Dosage	43.62	<0.001	Significant
	Geometry × Dosage	5.83	0.002	Significant

## Data Availability

The original contributions presented in this study are included in the article. Further inquiries can be directed to the corresponding author.
